# Extended spectrum β-lactamase-producing Enterobacterales in live and dead birds from rural poultry farms and urban live bird markets of Bangladesh

**DOI:** 10.3389/fmicb.2025.1560890

**Published:** 2025-07-17

**Authors:** Mohammed Badrul Amin, Kazi Injamamul Hoque, Ajrin Sultana Sraboni, Omar Faruk Bhuiyan, Tanjin Tamanna Happy, Munirul Alam, Dinesh Mondal, Mohammad Aminul Islam

**Affiliations:** ^1^Laboratory of Food Safety and One Health, Nutrition Research Division, icddr, b, Dhaka, Bangladesh; ^2^Infectious Disease Division, icddr, b, Dhaka, Bangladesh; ^3^Paul G. Allen School for Global Health, Washington State University, Pullman, WA, United States

**Keywords:** ESBL, Enterobacterales, live bird markets, rural poultry farms, dead birds, live birds, poultry

## Abstract

**Introduction:**

Poultry raised with antibiotic prophylaxis are significant reservoirs of extended-spectrum β-lactamase-producing Enterobacterales (ESBL-E). Improper disposal of poultry wastes is common in developing countries, increasing the risk of spreading ESBL-E. Previous studies largely focused on ESBL-producing *E. coli* in poultry, overlooking other Enterobacterales and dead birds.

**Methods:**

Between December 2019 and June 2021, this study investigated 220 fecal samples collected from 55 freshly slaughtered and 55 dead birds in urban live bird markets and rural poultry farms in Bangladesh for detection and enumeration of ESBL-E.

**Results:**

Overall 68% (*n* = 150) samples were positive for ESBL-E, with a significantly higher prevalence in urban live-bird markets (88%, *n* = 97) than rural poultry farms (48%, *n* = 53; *p* < 0.01, OR = 7.25, 95% CI: 3.77–14.71) and slightly higher in dead (*n* = 81, 74%) than live birds (*n* = 69, 63%). ESBL-producing *E. coli* was most common (66%, *n* = 146), followed by ESBL-producing *Klebsiella pneumoniae* (10%, *n* = 22), *Raoultella terrigena* (3%, *n* = 7) and *Enterobacter* spp. (3% *n* = 6). The abundance of ESBL-producing *E. coli* was significantly higher in urban live-bird markets than rural poultry farms (3.9 vs. 2.0 log_10_ CFU/g; *p* < 0.001, Cliff's Delta = 0.53, 95% CI: 0.40–0.65) and in dead than live birds (3.4 vs. 2.2 log_10_ CFU/g; *p* < 0.01, Cliff's Delta = 0.23, 95% CI: 0.08–0.38). The abundance of ESBL-producing *K. pneumoniae, Enterobacter*, and *R. terrigena* (1.6–1.8 log_10_ CFU/g) showed no significant difference between urban live-bird markets and rural poultry farms or between live and dead birds. A higher proportion of ESBL-E from urban live bird markets were resistant to 10 out of 11 antibiotic classes, compared to those from rural poultry farms (*p* < 0.05). Further, ESBL-E isolates from dead birds showed higher resistance to aminoglycosides, glycylcyclines, and penicillins+β-lactamase inhibitors than isolates from live birds (*p* < 0.05). Overall, 65% of isolates were resistant to penicillins, fluoroquinolones, and monobactams, while 2% were carbapenem-resistant. The prevalence of multi-drug resistant *E. coli* was higher in urban live bird markets (86%, *n* = 95) than rural poultry farms (45%, *n* = 49; *p* < 0.01). Among 181 ESBL-E, *bla*_TEM_ (62%, *n* = 114) was the most prevalent, followed by *bla*_CTX − M−group_ (17%, *n* = 32) and *bla*_SHV_ (12%, *n* = 22).

**Discussion:**

The widespread ESBL-E in poultry underscores the urgent need for improved biosecurity and waste management across the poultry supply chain.

## Highlights

- About 68% of poultry fecal samples contained ESBL-producing Enterobacterales (ESBL-E).
- The prevalence of ESBL-E was significantly higher in poultry from urban live bird markets compared to rural poultry farms (*p* < 0.05).
- The fecal load of ESBL-E was higher in dead birds compared to live birds.
- About 98% of ESBL-E were multi-drug resistant, with a significantly higher prevalence in dead birds.

## Introduction

The presence of extended-spectrum β-lactamase (ESBL) producing Enterobacterales in poultry has been a great concern, particularly in areas where humans live in close proximity to chickens. These bacteria are often enriched in the chicken gut due to prophylactic use of antibiotics during poultry production. This practice can increase the risk of transmission of antibiotic resistant bacteria to humans through direct contact with chicken. Indirect transmission of these bacteria can also occur via environmental contamination due to weak regulations in farm biosecurity, waste management practices, and poor water, sanitation and hygiene conditions (Magnusson et al., 2021). A compounding concern is that Enterobacterales species, which frequently acquire resistance to multiple antibiotics, are commonly present in both human and poultry gut microbiota. The overlapping species of Enterobacterales that cause infections in both humans and poultry include *E. coli, Klebsiella pneumoniae, Enterobacter* spp., and *Salmonella enterica* (Apata, 2009; Davies and Davies, 2010; Rehman et al., 2007). While *E. coli* is considered a normal gut flora of all warm-blooded animals, a small proportion of this organism can be pathogenic to both humans and poultry. In humans, *E. coli* cause intestinal infections such as diarrhea and dysentery, as well as extraintestinal infections including the urinary tract, central nervous system, skin, and soft tissue infections (Tenaillon et al., 2010; Alharbi et al., 2019). In poultry, it causes colibacillosis. Similarly, *Klebsiella*, an opportunistic pathogen, is known to cause severe nosocomial infections such as pneumonia, septicemia, and urinary tract infections (Fielding et al., 2012). *Enterobacter*, another member of Enterobacterales, is associated with a range of serious infections in humans. These include infections in the bloodstream, urinary tract, skin and soft-tissue, lower respiratory tract, intra-abdomen, central nervous system, heart, bones, joints, and eyes (Gaston, 1988). All these infections become difficult to treat when caused by multi-drug resistant (MDR) organisms especially, ESBL-producing bacteria (Davies and Davies, 2010; Levy and Marshall, 2004).

In Bangladesh, poultry sector contributes to 37% of the total animal protein consumption (Hamid et al., 2017). To meet this growing demand, poultry farming has been intensified often with poor regulatory oversights of antibiotic use. Many reports indicate that antibiotics are widely used not just to treat infections but also for prophylaxis and growth promotion in both small- and large-scale commercial farms across the country (Rahman et al., 2020; Hasan et al., 2011; Hassan et al., 2021; Mandal et al., 2022). Among the commonly used antibiotics were tetracyclines, ciprofloxacin, cephalosporin, colistin, and sulfonamides with trimethoprim, gentamicin, and erythromycin. Most of these antibiotics are also frequently used in humans and thus emergence of resistant bacteria in poultry increases the risk for human health (Islam et al., 2023a).

Over the years, poultry production in Bangladesh has become more concentrated and has expanded significantly across the country. While large-scale commercial poultry industries adhere to biosecurity measures and have improved waste management systems, small and medium-scale farms often lack such practices. Ironically, these small and medium-scale operators contribute the most to meet the country's demand for meat and eggs. Despite substantial growth in the poultry industry in Bangladesh, poultry waste management remains an underappreciated issue. In our previous study, we observed that poultry feces and carcasses are routinely disposed of or thrown near poultry sheds, where dogs and foxes scavenge for food (Alam et al., 2019). Dead or sick birds serve as an important source of pathogenic bacteria. A previous study reported that 36% of 279 dead or sick birds collected from different commercial poultry farms in Bangladesh were diagnosed as avian colibacillosis caused by pathogenic *E. coli* (Hasan et al., 2011). Around 56% of *E. coli* isolates were resistant to one or more antibiotics and 37% of the isolates were MDR. Interestingly, this study did not find any isolate producing extended spectrum β-lactamases. This study used samples collected during the period of 2008–2009, when the prevalence of ESBL-producing organisms in humans and animals were low.

In recent years, several studies have investigated antibiotic-resistant bacteria in poultry, but most have primarily focused on ESBL-producing *E. coli*, potentially underestimating other ESBL-producers within Enterobacterales (Islam et al., 2023a; Ilyas et al., 2021). Furthermore, no recent studies have assessed the prevalence of ESBL-producing Enterobacterales in dead birds. This study fills the knowledge gap by providing an updated estimate of these organisms in both dead and live birds, emphasizing the risk of their spillover to other animals, including scavengers, and their potential spread into surrounding environments closely linked to human activities.

## Materials and methods

### Study site and sample collection

**Figure 1:**
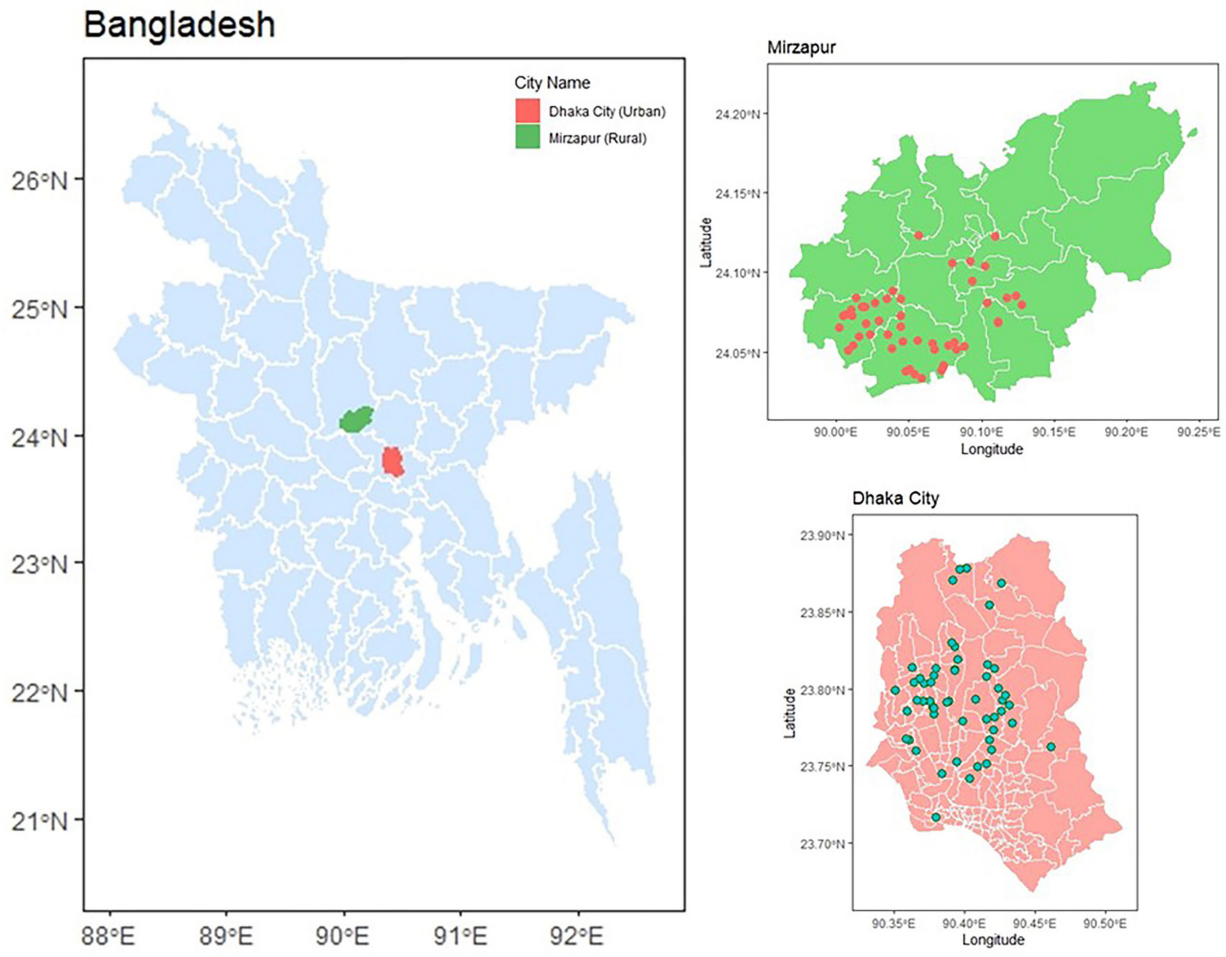
Sampling location of 55 live bird markets in Dhaka and 55 rural poultry farms in Mirzapur. The red and green dots on the map represent rural poultry farms and live bird markets, respectively.

We selected 55 urban live bird markets (LBM) in high-density residential areas in Dhaka city and 55 rural poultry farms (RPF) with traditional animal husbandry adjacent to households in rural Mirzapur, Tangail during January 2020–June 2021 (Figure 1). These locations were chosen because we had previously conducted a comprehensive antimicrobial resistance surveillance in humans, poultry, and the environment in the same areas (Rousham et al., 2021). This allowed us to leverage our established connections with vendors and farm owners, facilitating sample collection and data acquisition. The markets and farms, each selling broiler and layer chickens, were randomly selected to ensure representation of all LBM in Dhaka city and RPF in Mirzapur sub-district. The distance between LBM and RPF is ~55–65 km. From each LBM and RPF, we collected two types of samples: freshly slaughtered chickens (*n* = 110) and chickens that had died naturally within 3–5 h (*n* = 110). Since this was an exploratory study, we did not follow the standard sample size estimation and included all farms and markets that were part of our previous study. Both types of samples were collected on the same day from the same vendor of the market or farm. The dead and freshly slaughtered whole carcass was kept in separate sterile plastic bags after defeathering and transported to the laboratory within 4–6 h of collection maintaining cold chain (4–8°C).

### Sample processing, ESBL-producing bacteria isolation, and identification

To make an initial suspension, we took ~25 g of fecal materials from the cecum and small intestine into a labeled sterile whirl-pack bag, added 225 mL of peptone water (PW) and mixed homogeneously using a homogenizer (Stomacher^®^ 400 Circulator, Seward Ltd. UK). We used the suspension for serial dilution to obtain countable colony forming unit per gram (CFU/g) of the fecal material.

For ESBL-E culture, we prepared a 10-fold serial dilution of the initial suspension in PW and inoculated 100 μl of the last two dilutions (10^−2^ and 10^−3^) on the CHROMagar ESBL plate (CHROMagar, Paris, France) by spread plating and incubated the plates at 37°C for 18–24 h. We identified pink-colored colonies as *E. coli*, and metallic blue colonies as *Klebsiella, Enterobacter*, or *Citrobacter* spp. following the manufacturer's instructions (CHROMagar, Paris, France). We selected a single colony of each color type from each plate for species confirmation using the API 20E test (bioMérieux, Marcy l'Etoile, France). After confirmation, we quantified ESBL-producing *E. coli, Klebsiella* or *Enterobacter* isolates as CFU/g of feces, and stored a representative isolate of each species in glycerol broth at −20°C for further analysis.

For *Salmonella* spp., we took 1.0 mL of the initial PW suspension, added to 9 mL of Buffered Peptone Water (BPW) in a sterile falcon tube and incubated it overnight at 37°C in aerobic condition. We transferred 1 mL of BPW suspension to 10 mL of MKTTn (Muller-Kauffmann Tetrathionate Novobiocin) broth, supplemented with iodine-iodide solution and Novobiocin, and incubated at 37°C for 18–24 h. Additionally, we mixed 0.1 mL of BPW suspension to RVS (Rappaport Vassiliadis Soya) broth and incubated at 42°C for 24 h. We inoculated a loopful of the overnight enrichment culture from both MKTTn and RVS broths onto both XLD (Xylose Lysine Deoxycholate) and BGA (Brilliant Green Agar) plates and incubated at 37°C for 24 h. We considered red-colored colonies on BGA plates and red colonies with/without a black center on XLD plates as presumptive colonies of *Salmonella* spp. We tested one or two presumptive colonies using the ONPG (o-nitrophenyl-β-D-galactopyranoside) biochemical tests (Sigma-Aldrich, Missouri, USA). Colonies that showed a white color (indicating a negative result) in the ONPG test were further confirmed by PCR for the *invA* gene (Rahn et al., 1992). We used *Salmonella Typhimurium* ATCC 14028 and *E. coli* ATCC 25922 strains as positive and negative controls, respectively.

### Confirmation of ESBL production and antibiotic susceptibility testing

We confirmed all presumptive ESBL-producing bacterial isolates obtained from CHROMagar ESBL plates for ESBL production using a combined disk test with cefotaxime-clavulanate 30/10 μg and ceftazidime-clavulanate 30/10 μg disks, following the Clinical and Laboratory Standards Institute (CLSI) guidelines (CLSI, 2022). We performed antibiotic susceptibility of the ESBL-producing isolates against 11 different classes of antibiotic, including amoxicillin-clavulanic acid (30 μg), aztreonam (30 μg), cefoxitin (30 μg), tigecycline (15 μg), ampicillin (10 μg), gentamicin (10 μg), tetracycline (30 μg), meropenem (10 μg), ciprofloxacin (5 μg), trimethoprim-sulfamethoxazole (25 μg), and chloramphenicol (30 μg) using disk diffusion test according to CLSI guidelines. We classified the isolates as resistant and sensitive to antibiotics based on the CLSI criteria (Patel, 2017). An isolate showing resistance to at least one antibiotic from three or more different classes was classified as MDR (Amin et al., 2021).

### PCR for ESBL encoding genes

We extracted genomic DNA from isolated bacterial colonies using the boiling method (Mahmud et al., 2018). Briefly, 1–2 single colonies were added to a microcentrifuge tube containing 300 μL of distilled water, boiled for 10 min in a water bath, and then centrifuged at 12,000 rpm for 5 min. The resulting supernatant was used as a DNA template for PCR to identify the presence of common ESBL-encoding genes, including *bla*_TEM_, *bla*_SHV_, and *bla*_CTX − M−group − 1, −2, −8, −9, and − 25_, using the primer sequences and PCR conditions described previously (Amin et al., 2020).

### Statistical analysis

We conducted data cleaning and statistical analyses using STATA (version 15.0SE, Stata Corporation, College Station, Texas, USA), while data visualization was performed using R programming language (Wickham et al., 2019). We imputed zero (negative) values of CFU/g counts, with a randomly generated number between zero and the limit of detection for each sample, assuming a normal distribution following recommended methods for left-censored data (Canales et al., 2018). We used chi-square tests and calculated odds ratios (OR) with 95% confidence intervals (CI) to examine variations in the prevalence of ESBL-E across different locations and bird types. To assess differences in bacterial abundance, we conducted Mann-Whitney U tests, followed by effect size estimation using Cliff's Delta with 95% CI to quantify the magnitude of the observed differences.

## Results

### Prevalence and abundance of ESBL-producing bacteria

**Figure 2:**
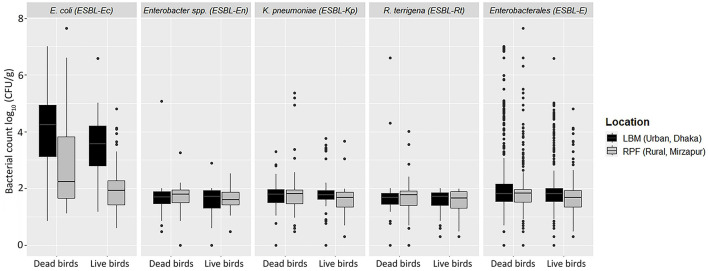
Abundance of ESBL-E including *E. coli, K. pneumoniae, Enterobacter* spp., and *Raoultella terrigena* in live and dead birds from live bird markets and rural poultry farms.

We identified four bacterial species within Enterobacterales including *E. coli, Klebsiella pneumoniae, Enterobacter* spp., and *Raoultella terrigena*. Among the 220 fecal samples collected from LBM and RPF, 68% (*n* = 150) tested positive for ESBL-producing Enterobacterales (ESBL-E), with a median count of 1.8 [IQR 0.5] log_10_ CFU/g of bird feces (Table 1; Figure 2). The prevalence of ESBL-E was significantly higher in LBM (*n* = 97, 88%) compared to RPF (*n* = 53, 48%; *p* < 0.01, OR = 7.25, 95% CI: 3.77–14.71) and was also slightly higher in dead birds (*n* = 81, 74%) than in live birds (*n* = 69, 63%). However, the median bacterial load of ESBL-E was similar between live and dead birds (Figure 2).

**Table 1:** Prevalence of ESBL-E in live and dead birds from live bird markets and rural poultry farms, and distribution of β-lactamase encoding genes in ESBL-E isolates.

**ESBL-producing bacteria**	**LBM (urban, Dhaka)**		**RPF (rural, Mirzapur)**		**Gene encoding** β**-lactamase in ESBL-E (*****n*** = **181)**				
	**Live birds (*n* = 55)**	**Dead birds (*n* = 55)**	**Live bird (*n* = 55)**	**Dead bird (*n* = 55)**	***bla*_TEM_**	***bla*_SHV_**	***bla*_CTXM − 1_**	***bla*_CTXM − 2_**	***bla*_CTXM − 9_**
	***n*(%)**	***n*(%)**	***n*(%)**	***n*(%)**	***n*(%)**	***n*(%)**	***n*(%)**	***n*(%)**	***n*(%)**
Enterobacterales	48 (87)^*^	49 (89)^*^	21 (38)	32 (58)	113 (62)	22 (12)	30 (17)	1 (0.6)	1 (0.6)
*E. coli*	48 (87)^*^	48 (87)^*^	20 (36)	30 (55)	104 (71)	10 (7)	19 (13)	1 (0.7)	1 (0.7)
*K. pneumoniae*	7 (13)	6 (11)	2 (4)	7 (13)	6 (27)	9 (41)	7 (32)	0	0
*Enterobacter* spp.	1 (2)	2 (4)	1 (2)	2 (4)	1 (17)	1 (17)	2 (33)	0	0
*R. terrigena*	1 (2)	2 (4)^#^	0 (0)	4 (7)^#^	2 (29)	2 (29)	2 (29)	0	0

^*^Significantly high by location (*p* < 0.01) and ^#^Significantly high according to bird status (*p*= 0.05).

Among the ESBL-E isolates, *E. coli* (ESBL-Ec) was the most prevalent (66%, *n* = 146) species, with a median count of 3.0 [IQR 2.3] log_10_ CFU/g (Table 1; Figure 2). The prevalence of ESBL-Ec was significantly higher in LBM (*n* = 96, 87%) than in RPF (*n* = 50, 46%; *p* < 0.01, OR = 6.67, 95% CI: 3.70–12.50). In RPF, ESBL-Ec was detected in 55% (*n* = 30) of dead birds and 36% (*n* = 20) of live birds, whereas in LBM, the prevalence remained consistent in both live and dead birds (87%, *n* = 48). The median count of ESBL-Ec was significantly higher in LBM (3.9 [IQR 1.7] log_10_ CFU/g) compared to RPF (2.0 [IQR 1.5] log_10_ CFU/g; *p* < 0.001, Cliff's Delta = 0.53, 95% CI: 0.40–0.65) and in dead birds (3.4 [IQR 2.7] log_10_ CFU/g) compared to live birds (2.6 [IQR 2.1] log_10_ CFU/g; *p* < 0.01, Cliff's Delta = 0.23, 95% CI: 0.08–0.38).

*K. pneumoniae* (ESBL-Kp) was the second most frequently detected ESBL-producing species, found in 10% (*n* = 22) of samples, with a median count of 1.8 [IQR 0.5] log_10_ CFU/g (Table 1; Figure 2). The prevalence of ESBL-Kp in live birds was higher in LBM (13%, *n* = 7) compared to RPF (4%, *n* = 2), while in dead birds, it was 11% (*n* = 6) in LBM and 13% (*n* = 7) in RPF. The median count of ESBL-Kp remained consistent across live and dead birds in both settings, ranging from 1.7 to 1.8 [IQR 0.4–0.6] log_10_ CFU/g of feces (Figure 2).

Among the remaining ESBL-producing species, *R. terrigena* (ESBL-Rt) was detected in 3.2% (*n* = 7) of samples, with a median count of 1.7 [IQR 0.5] log_10_ CFU/g of feces (Table 1; Figure 2). Although its prevalence was slightly higher in dead birds (5.4%) than in live birds (0.9%; *p* = 0.05), the median count of ESBL-Rt was similar in both groups, ranging 1.6–1.7 [IQR 0.4–0.5] log_10_ CFU/g of feces (Figure 2).

*Enterobacter* spp. (ESBL-En), the least frequently detected ESBL-producing species, was found in 2.7% (*n* = 6) of samples, with a median count of 1.7 log_10_ CFU/g of feces (Table 1; Figure 2). While ESBL-En did not differ significantly between LBM and RPF, a relatively higher number of dead bird samples (4%, *n* = 4) tested positive compared to live bird samples (2%, *n* = 2). the median count of ESBL-En was in the range of 1.7–1.8 (IQR 0.5–0.6) log_10_ CFU/g of fecal samples from live and dead birds.

*Salmonella* spp. was detected in 10% (*n* = 23) of samples, with a significantly higher prevalence in LBM (15%, *n* = 16) compared to RPF (6%, *n* = 7; *p* = 0.05). Dead birds were also more frequently positive for *Salmonella* (17%, *n* = 19) than live birds (4%, *n* = 4; *p* < 0.01). However, none of the *Salmonella* isolates tested positive for ESBL.

### Prevalence of ESBL gene types

Among the 181 ESBL-E isolates obtained from 150 fecal samples, 133 (73%) carried at least one of the three major ESBL genes: *bla*_TEM_, *bla*_SHV_, and *bla*_CTX − M_. The predominant species, ESBL-Ec, harbored *bla*_TEM_ most frequently (71%, *n* = 104), followed by *bla*_CTX − M−1_ (13%, *n* = 19), *bla*_SHV_ (7%, *n* = 10), *bla*_CTX − M−2_ and *bla*_CTX − M−9_, each detected in 1% of the isolates. Meanwhile, in ESBL-Kp, *bla*_SHV_ was the most prevalent gene (41%, *n* = 9), followed by *bla*_CTX − M−1_ (32%, *n* = 7), and *bla*_TEM_ (27%, *n* = 6). Among ESBL-En, *bla*_CTX − M−1_ was present in 33% (*n* = 2) of isolates, while *bla*_TEM_ and *bla*_SHV_ were each detected in single isolates. Notably, 29% (*n* = 2) of the ESBL-Rt isolates carried *bla*_CTX − M−1_, *bla*_TEM_ and *bla*_SHV_ (Table 1). None of the ESBL-E isolates in this study carried *bla*_CTX − M−8_ and *bla*_CTX − M−25_.

### Antibiotic susceptibility testing of ESBL-E

**Figure 3:**
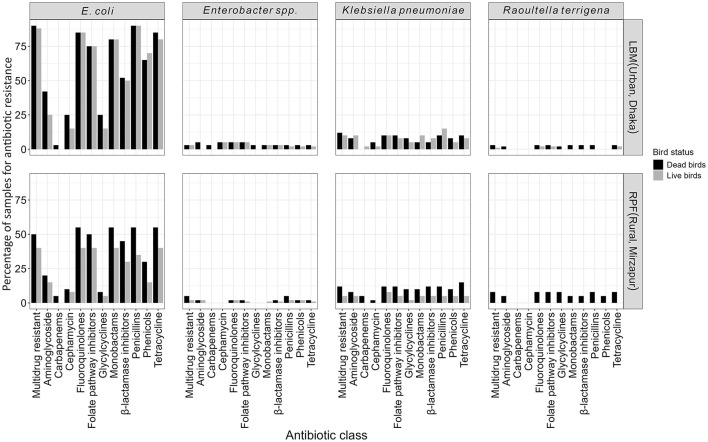
Antibiotic susceptibility patterns of ESBL-E (*E. coli, K. pneumoniae, Enterobacter* spp., and *R. terrigena*) isolates categorized by their sources and viability status of the birds.

A significantly higher proportion of ESBL-E from LBM exhibited resistance to 10 of the 11 antibiotic classes, except for carbapenems, compared to those from RPF (*p* < 0.05). Additionally, resistance to aminoglycosides, glycylcyclines, and penicillins+β-lactamase inhibitors was significantly more prevalent in ESBL-E from dead birds compared to those from live birds (*p* < 0.05). Around 65% of ESBL-Ec isolates were resistant to fluoroquinolones, and monobactams, while 15% were resistant to cephamycins and glycylcyclines, and only 2% of the isolates were resistant to carbapenems (Figure 3).

Nearly all ESBL-E isolates (99%, *n* = 179) were MDR, with the majority being *E. coli* (80%, *n* = 144), followed by *K. pneumoniae* (12%, *n* = 21), *Enterobacter* spp. (4%, *n* = 7), and *R. terrigena* (4%, *n* = 7). MDR *E. coli* was significantly more prevalent in birds from LBM (86%, *n* = 95) compared to the birds from RPF (45%, *n* = 49; *p* < 0.01; Figure 3). The overall prevalence of MDR *K. pneumoniae* and *Enterobacter* spp. was around 10% (*n* = 21) and 3% (*n* = 7), respectively, with no significant differences between LBM and RPF or between dead and live birds. Notably, we only found MDR *R. terrigena* in dead birds (7%, *n* = 4) from RPF but not any from live bird samples (Figure 3).

**Figure 4:**
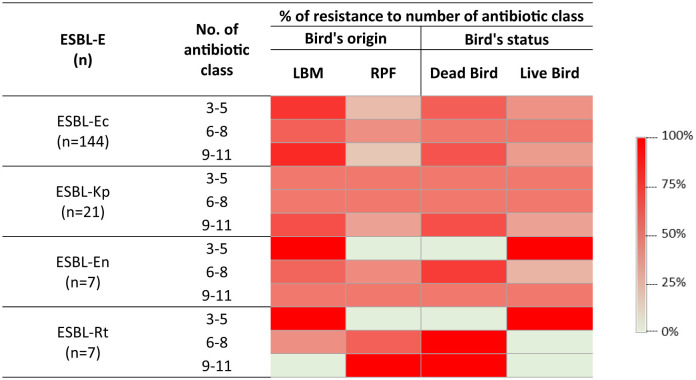
Heatmap showing the distribution of resistance to multiple antibiotic classes among ESBL-E isolates, categorized by their sources and viability status of the birds. *n*, number of species; ESBL-Ec, ESBL-producing *E. coli*; ESBL-Kp, ESBL-producing *K. pneumoniae*; ESBL-En, ESBL-producing *Enterobacter* spp.; ESBL-Rt, ESBL-producing *R. terrigena*.

Further analysis of ESBL-E isolates revealed that ~67% (*n* = 121) of isolates were resistant to 6–8 antibiotic classes. *E. coli* was the most prevalent accounting for 60% of isolates, followed by *Enterobacter* spp. (58%), *K. pneumoniae* (50%), and *R. terrigena* (40%) (Figure 4). The extent of resistance was even more pronounced in isolates from LBM where 50–83% of ESBL-E except *R. terrigena* were resistant to more than eight antibiotic classes compared to those from RPF (17–50%) (Figure 4). A similar pattern was observed in dead birds (50–67%), where resistance to more than eight antibiotic classes was highly prevalent compared to live birds (33–50%). Among these isolates, all ESBL-producing *R. terrigena*, 67% of *K. pneumoniae*, 65% of *E. coli*, and 50% of *Enterobacter* spp. isolates from dead birds were resistant to more than eight antibiotic classes.

## Discussion

Our study found a 68% prevalence of ESBL-E in poultry feces, with a predominance of ESBL-Ec. The prevalence was significantly higher in samples from LBM in Dhaka (88%) compared to RPF in Mirzapur (48%). However, there was no significant difference in the abundance of ESBL-E between these two settings. We observed a higher prevalence of ESBL-E and *Salmonella* spp. in dead birds compared to live birds, with the difference being particularly pronounced in birds from RPFs, but not in the LBMs. In poultry farms, sick birds are typically isolated and given antibiotic treatment, which may result in a higher abundance of resistant organisms in their gut compared to healthy birds on the same farm. In contrast, poultry in LBMs are held for a short period, and vendors usually do not separate sick birds from healthy ones, leading to similar level of ESBL-E status in both live and dead birds.

Our observed prevalence is considerably higher than the pooled estimates from a meta-analysis conducted in Bangladesh, which reported an ESBL-Ec prevalence of 17% (95% CI: 11–23%) in humans and 22% (95% CI: 9–34%) in animals (Islam et al., 2023b). Comparisons with neighboring countries further highlight the regional burden of antimicrobial resistance in poultry. A study in India found that the prevalence of ESBL-producing bacteria in animals ranged from 12 to 33% between 2013 and 2019. In Nepal, the prevalence of ESBL-E in rural poultry farms was around 30%, slightly lower than the observed prevalence (38%) in RPF in our study (Hosuru Subramanya et al., 2020). Similarly, a study in Philippines reported a 67% prevalence of ESBL-Ec in birds from urban areas, which is lower than the observed prevalence of ESBL-Ec (87%) in LBM in our study (Ievy et al., 2020). Notably, all these studies focused primarily on the prevalence of ESBL-E in live poultry, with no data on dead poultry.

Overall, the abundance of ESBL-Ec was high, with a median count of 3.0 [IQR 2.3] log^10^ CFU/g of feces, and was significantly higher in dead birds compared to live birds. Typically, the abundance of ESBL-E is not estimated in surveillance studies. In our previous study, we estimated the abundance of ESBL-Ec in humans, poultry, and interfacing environments to better understand transmission dynamics. In areas with a high overall prevalence of ESBL-Ec, bacterial count data offers more accurate estimates of the contribution of different sources to transmission and helps assess the underlying risk of environmental contamination. Although establishing a threshold for acceptable levels of ESBL-Ec in the environment may not be feasible in resource-limited settings at this time, future interventions aimed at improving WASH in these regions could incorporate this approach.

The higher abundance of ESBL-Ec in dead birds might be linked to infections, such as colibacillosis, caused by avian pathogenic *E. coli* (Ievy et al., 2020). In such infectious events, farmers use antibiotics more aggressively for treatment of the sick bird as well as prophylactically to prevent the further spread of infections within the flock (Chowdhury et al., 2022). The indiscriminate use of antibiotics drives the selection and overgrowth of antibiotic-resistant bacteria in poultry gut. Additionally, infections compromise bird's immune system, reducing its ability to suppress microbial overgrowth and facilitating resistant strains to proliferate. In this study, we collected carcasses within a few hours of death of the bird to ensure that the observed bacterial growth was not solely due to natural post-mortem overgrowth. However, even if such overgrowth occurs, it still poses a significant public health concern due to the widespread practice of open disposal of poultry waste, including carcasses, into the nearby environment, where scavengers such as dogs, crows, rats, and foxes feed on them (Alam et al., 2019). This practice likely increases the risk of resistant organisms spilling over into other animals and the surrounding human habitat.

The prevalence of ESBL-Kp in poultry in Bangladesh has not been extensively studied, despite the recent classification of *K. pneumoniae* as a priority pathogen by the WHO (World Health Organization, 2024). In our study, we observed a 10% prevalence of ESBL-Kp. A previous study reported a 20% prevalence in the Noakhali region by analyzing only 12 samples, and did not confirm whether the *K. pneumoniae* isolates were ESBL-producers (Munim et al., 2024). While the rate may seem relatively low, continuous monitoring of this opportunistic pathogen is crucial, as ESBL-Kp is a leading cause of mortality worldwide (Murray et al., 2022). *R. terrigena* is an emerging pathogen, particularly in pediatric clinical settings. This study is the first to report a 3% prevalence of ESBL-producing *R. terrigena* in poultry in Bangladesh. The detection of this organism in poultry raises concerns about the potential zoonotic transmission and its implications for public health (Aissaoui et al., 2021).

Consistent with previous findings in Bangladesh, the prevalence of *Salmonella* spp. was significantly higher in dead birds compared to live birds, likely due to salmonellosis caused by certain *Salmonella* serovars, a major concern for the poultry industry (Mahmud et al., 2011). In this study, around 10% of samples tested positive for *Salmonella* spp., but none were ESBL-producers. The rate is lower than that reported in other studies from Bangladesh, where the prevalence ranged from 20% to as high as 75–80%, with a substantial proportion of isolates being ESBL-producers (Mahmud et al., 2011; Talukder et al., 2020; Parvin et al., 2022). These discrepancies might be attributed to differences in farming practices, antibiotic uses, and biosecurity measures. The substantial differences in prevalence and antibiotic resistance highlight the need for a systematic investigation to understand the true burden of *Salmonella* spp. in poultry.

The rapidly growing poultry industry in Bangladesh plays a crucial role in providing high-quality animal protein. However, the indiscriminate use of antibiotics in poultry production, often without active oversight, may contribute to the emergence of MDR bacteria. This resonates with the findings of our study that 98% of ESBL-producing bacteria were identified as MDR (Masud et al., 2020). Even more concerning is the reported use of banned antibiotics like colistin sulfate throughout the poultry production cycle (Masud et al., 2020). Surprisingly, over 95% of farmers administer antibiotics without veterinary supervision, further exacerbating the risk of resistance development (Shamsuzzaman et al., 2007). The alarmingly high prevalence of MDR bacteria in poultry in Bangladesh is not an emerging issue. A recent systematic review reported that 93% of bacteria isolated from poultry across the country was identified as MDR (Islam et al., 2023a). The presence of MDR bacteria in poultry poses a serious public health risk, as these bacteria can be transmitted to humans through the direct contact of contaminated food, and cross-contamination during handling and processing of poultry (Rousham et al., 2021). Additionally, poultry feces and dead birds disposed to the environment can contaminate soil and water bodies, further facilitating the spread of antibiotic-resistant bacteria.

We found that a significant proportion of ESBL-producing bacteria from LBM exhibited resistance to more than eight antibiotic classes highlighting concerns about the emergence of extensively drug-resistant foodborne bacteria. This finding is consistent with our previous study, although the underlying causes remain unclear. One possible explanation is that LBM in Dhaka receive chicken from various parts of the country, showing greater variability in ESBL-E colonization. In contrast, poultry within the same farm are exposed to a uniform environmental condition, resulting in lower variability. Additionally, poultry transported to LBM often experience stress and are given prophylactic antibiotics, which may account for the higher resistance observed in isolates from LBM compared to those from RPF. Furthermore, the overcrowded conditions and poor hygienic practices in poultry slaughtering areas create an ideal environment for the spread of MDR organisms (Sayeed et al., 2017).

Among the frequently reported β-lactamase genes, *bla*_TEM_ was the most prevalent, detected in 62% of ESBL-E isolates. The finding aligns with previous studies in Bangladesh, as well as in other countries like China and Portugal, highlighting its widespread distribution across different geographical regions (Parvin et al., 2022). In addition, ~18% of ESBL-Ec isolates carried *bla*_CTX − M_ including *bla*_CTX − M−1_, *bla*_CTX − M−2_ and *bla*_CTX − M−9_. However, this result contrasts with a prior study in Bangladesh, where no *bla*_CTX − M_ was found in any ESBL-Ec isolates (Parvin et al., 2022). Interestingly, two other studies in Bangladesh reported that over 90% of ESBL-Ec isolates from chicken feces and households water samples harbored the *bla*_CTX − M−1_ (Hasan et al., 2012; Talukdar et al., 2013), demonstrating significant variations in resistance gene prevalence across different studies. These discrepancies might be due to variations in sample sources, geographic locations, and antibiotic usage patterns in poultry farming accentuating the need for a continuous surveillance of antimicrobial resistance in these settings.

One limitation of this study is that the prevalence of ESBL-E was estimated based on 220 poultry samples from only two settings, limiting the generalizability of the findings to the broader area. Furthermore, the study relied on a culture-based approach using chromogenic agar media for estimating the prevalence and abundance of ESBL-E in poultry. This methodology could result in an underestimation of the actual prevalence of ESBL-E, potentially failing to capture the broader epidemiological trends across the country. However, the screening and phenotypic confirmation methods used in this study could serve as a valuable tool for routine surveillance of the poultry production and supply chain. This method enables regulatory authorities to closely monitor the presence of ESBL-producing bacteria, which can help inform strategies to control the spread of antibiotic resistance in poultry farming.

In conclusion, this study demonstrates that both live and dead birds serve as reservoirs for multi-drug resistant ESBL-producing bacteria, with contamination levels notably higher in LBM than in RPF. The widespread presence of these resistant bacteria, combined with poor hygienic practices, inadequate biosecurity and improper poultry waste management, presents a significant public health threat. Given the close interactions between humans and poultry in both urban and rural settings, effective waste management including proper disposal of dead poultry, improved hygiene practices and antibiotic stewardship throughout poultry production and supply chain, are crucial for reducing the spread of antibiotic-resistant bacteria.

## Data Availability

The raw data supporting the conclusions of this article will be made available by the authors, without undue reservation.
